# N-*trans*-Feruloyloctopamine Wakes Up BBC3, DDIT3, CDKN1A, and NOXA Signals to Accelerate HCC Cell Apoptosis

**DOI:** 10.1155/2021/1560307

**Published:** 2021-05-22

**Authors:** Bin Ma, Jing Li, Wen-Ke Yang, Mei-Gui Zhang, Xiao-Dong Xie, Zhong-Tian Bai

**Affiliations:** ^1^Key Laboratory of Preclinical Study for New Drug of Gansu Province, School of Basic Medical Science, Lanzhou University, Lanzhou 730000, China; ^2^General Surgery Department, The First Hospital of Lanzhou University, The First Clinical Medical College, Lanzhou University, Lanzhou 730000, Gansu Province, China; ^3^Key Laboratory of Biological Therapy and Regenerative Medicine, Gansu Province, China

## Abstract

N-*trans*-Feruloyloctopamine (FO), a natural compound, was reported in our previous study to inhibit a tumor cell malignant phenotype by AKT- and EMT-related signals and might be used as a promising drug for HCC treatment. However, the specific targets and detailed mechanisms still need to be clarified. Screening with RNA-Seq in Huh7 cells treated with FO revealed that 317 genes were modulated, of which 188 genes were upregulated and 129 genes were downregulated. Real-time cell analyzer and flow cytometry data reveal that tumor cell proliferation and apoptosis were impacted by FO. DAVID bioinformatic data showed that most of the biological process GO terms are related to proliferation and apoptosis. KEGG enrichment analysis showed that FO mainly regulates PI3K-AKT- and apoptosis-related signals, in which BBC3, DDIT3, NOXA, and CDKN1A on the surface serve as the novel targets of FO inducing HCC cell apoptosis. The result implied that FO might exacerbate HCC cell apoptosis by regulating BBC3, DDIT3, CDKN1A, and NOXA signals. The obstacle effect of FO can provide new targets and new credibility for the treatment of liver cancer.

## 1. Introduction

Hepatocellular carcinoma (HCC) is the main malignant tumor in the world, which ranks fourth among tumor-related deaths. The incidence of HCC is rising with Asia alone contributing to more than 70% of incidence and mortality worldwide [1, 2]. Surgical resection is still the optimal strategy for early HCC. Advanced HCC still presents poor prognosis even if surgery, radiotherapy, and chemotherapy are given [3]. Chemotherapeutic drugs result in not only strong resistance but also more side effects [4].

**Figure 1:**
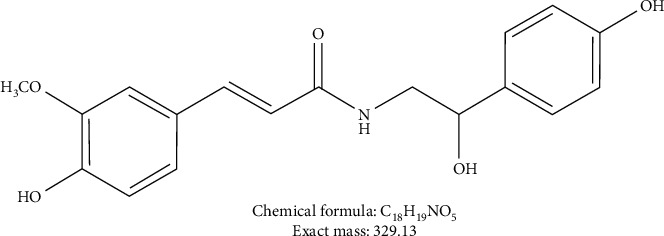
Molecular structure, chemical formula, and molecular weight of FO.

Natural products have gained attention for a long time in cancer therapy because of their strong efficacy and low toxicity, exhibiting a wide range of biological activities against a variety of diseases, such as infectious diseases and cancer [5]. N-*trans*-Feruloyloctopamine (FO) is the extract of garlic skin, which possesses high antioxidant activity and selectively induces cell apoptosis in leukemia tumors [6]. It might be considered as a promising anticarcinogen. Our previous report showed that FO inhibits HCC cell invasion via AKT-, mitogen-activated protein kinase- (MAPK-), and EMT-related signals [7]. The molecular structure of FO is shown in Figure 1, and the method of synthesizing FO was done according to our previous report. However, the drug targets and the detailed mechanisms have been masked. Herein, genome-wide transcriptome profiling (RNA-Seq) was used to screen the differentially expressed genes (DEGs) in FO-treated Huh7 cells, with the aim of uncovering the pharmacologic pattern. We hypothesized that FO might regulate related genes in apoptosis and PI3K-AKT pathways to mediate the proliferation and apoptosis of liver cancer cells.

## 2. Materials and Methods

### 2.1. Cell Lines

HCC cell lines Huh7 and Hep3B were purchased from the Cell Bank of the Chinese Academy of Sciences (Shanghai, China). HCC cell lines were maintained in high glucose DMEM (Invitrogen, California, USA) supplemented with 10% fetal bovine serum (FBS) (Life Technologies) and 1% (100 U/mL) penicillin-streptomycin (Life Technologies), then sustained at 37°C in a humidified incubator at 5% CO_2_.

### 2.2. Real-Time Analysis of Cell Proliferation

Real-time cell analysis (RTCA; Roche Applied Science, GmbH, Penzberg, Germany) experiments were carried out using the xCELLigence RTCA DP instrument. Briefly, 5 × 10^3^ cells were seeded in 16-well microtiter E-plates for the cell proliferation assay, and after incubating for 15 min, the baseline was detected. After the cells were incubated for 10 hours, FO (2.0 mM) and DMSO were added to the Huh7 and Hep3B cells, respectively, and then the electrical impedance in each well was measured continuously for nearly 90 hours. The slope indicates the increase in cell impedance per unit time (slope = impedance/time). The mean ± SD was calculated from three individual replicate wells.

### 2.3. Flow Cytometry Assay

When the cell fusion reached 60%, Huh7 and Hep3B cells were treated with FO and DMSO, respectively, and digested with EDTA-free trypsin after 24 hours. After the cells were collected, they were washed twice with cold PBS and resuspended with 1x binding buffer at a concentration of 1 × 10^6^ cells/mL. Then, 5 *μ*L of FITC Annexin V and 5 *μ*L of PI were added, and the cells were gently vortexed and incubated for 15 min at RT (25°C) in the dark. Finally, 400 *μ*L of 1x binding buffer was added, and the solution was analyzed by flow cytometry within 1 hour. Cell apoptosis was studied with the help of BD LSR II (Becton, Dickinson and Company, USA). Data were analyzed using FlowJo software (FlowJo v10; LLC, Ashland, OR, USA).

### 2.4. RNA-Seq Analyzer

Huh7 cells were treated with FO and DMSO, respectively, digested with TRIzol (Thermo Fisher Scientific) after 24 hours, and collected. The two samples were shipped to the GENEWIZ Company (http://www.genewiz.com/) for library construction and RNA-Seq. Sequencing library construction included RNA quality checking (Agilent 2100, Agilent Eukaryote Total RNA Nano Kit), library construction (Illumina TruSeq RNA Sample Prep Kit), library purification (AMPure XP beads, Beckman Coulter), insert fragment test (Agilent 2100, Agilent High-Sensitivity DNA Kit), quantitative analysis of library (Agilent Bioanalyzer 2100 and Qubit), and the cBOT Cluster Generation System (TruSeq PE Cluster Kit v4-cBot-HS). High-throughput sequencing was performed with TruSeq SBS Kit v4-HS (Illumina HiSeq 2000).

### 2.5. Differentially Expressed Genes and Bioinformatics Analysis

Differentially expressed genes were examined using the R/Bioconductor package edgeR and established by log2fold change (log2FC) and *P* value (|log2(FC)| > 1.00; *P* < 0.05). The gene set functional analysis and pathway analysis were analyzed using the DAVID bioinformatics tools. Gene Ontology (GO) and Kyoto Encyclopedia of Genes and Genomes (KEGG) pathway analysis were performed by setting all the GO terms and KEGG pathway genes as background genes.

The Pathview library was used to generate the “PI3K-AKT” and “Apoptosis” signaling pathways. The fold values of significantly changed genes were mapped by colors on native KEGG, where green represents downregulated expression and red represents upregulated expression levels in relation to the control group. In order to show a comprehensive image concerning the regulation of the analyzed signaling pathways, all genes whose expression was significantly different without a cut-off at fold values were visualized.

### 2.6. Real-Time Quantitative PCR Analysis

Four representative genes involved in the “PI3K-AKT” and “Apoptosis” signaling pathways were validated by Q-PCR in Huh7 treated with FO compared to NC. Total RNA was extracted using TRIzol (Thermo Fisher Scientific). Double-stranded cDNA was synthesized using the PrimeScript RT Reagent Kit (TaKaRa) according to the manufacturer's instructions. Subsequently, Q-PCR was performed using SYBR Premix Ex Taq™ II (TaKaRa). The 2^−ΔΔCT^ method was used to quantify the relative expression of each mRNA, using GAPDH as an internal control. All experiments were conducted in triplicate. Differences in mRNA expression between the groups were evaluated with a paired-sample *t*-test using the GraphPad Prism 5.0 software. It is considered significant when *P* < 0.05. Primer sequences are as follows: NOXA—forward: 5′-GTGCCCTTGGAAACGGAAGA-3′, reverse: 5′-CCAGCCGCCCAGTCTAATCA-3′; CDKN1A—forward: 5′-GGGATGTCCGT CAGAACCCA-3′, reverse: 5′-CACCCTCCAGTGGTGTCTCG-3′; PUMA—forward: 5′-CTGTGAATCCTGTGCTCTGC-3′, reverse: 5′-AATGAATGCCAGTGGTCACA-3′; CHOP—forward: 5′-GGAAACAGAGTGGTCATTCCC-3′, reverse: 5′-CTGCTTGAGCCGTTCATTCTC-3′; and GAPDH—forward: 5′-ATCTTCCAGGAGCGAGATCCC-3′, reverse: 5′-AGTGAGCTTCCC GTTCAGCTC-3′.

### 2.7. Statistical Analysis

Statistical analysis was performed with the SPSS 15.0 and GraphPad Prism 5.0 software. All tests were two-tailed; Student's *t*-test was used for statistical comparisons, and *P* < 0.05 was considered statistically significant.

## 3. Results

### 3.1. FO Exerted an Inhibitory Effect on Proliferative Activity of HCC Cells and Stimulated Apoptosis

**Figure 2:**
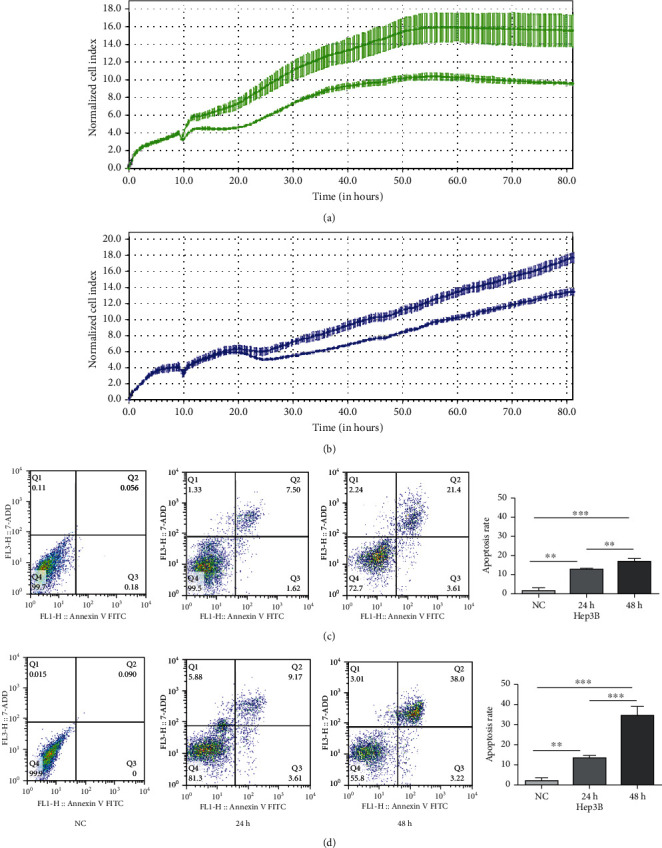
FO can inhibit cell proliferation and stimulate apoptosis in Huh7 cells. (a, b) Cell index values of Huh7 and Hep3B cells cultivated for 90 h. Electrical impedance was measured throughout the cultivation period at a 15 min frequency. Green and blue curves represent the proliferation of Huh7 and Hep3B cells treated with DMSO and FO, respectively. The mean cell index values with SE for the three repetitions in each group. (c, d) Hep3B and Huh7 cells were treated with DMSO for 24 hours, FO for 24 hours and 48 hours, and then stained with Annexin V and PI.

To deeply explore the function of FO, we performed Real-Time Cell Analysis (RTCA) and flow cytometry to detect cell proliferation and apoptosis, respectively. After intervention with FO in our previous study, HCC cells reached an inhibition corresponding to IC50 at a dose of approximately 2.00 mM. In order to avoid the effect of drug toxicity on cell viability, the concentration of FO in this study was kept consistently at 2.00 mM [7]. The experiment group is treated with FO, and the 0.2% DMSO-treated group is used as a control. To observe the effect of FO on Hep3B cells and Huh7 cell proliferation, changes in the cell index (CI) values were recorded on HCC cells treated with FO. RTCA data showed that a significant decrease in CI was observed after adding FO compared with DMSO (Figures 2(a) and 2(b)). Flow cytometry analysis after staining Huh7 and Hep3B cells with Annexin V-FITC/PI showed that the percentage of the apoptosis rate has a significant increase compared with NC (24-48 h) (Figures 2(c) and 2(d)). These data highlighted the antiproliferation and proapoptosis activity of FO.

### 3.2. Gene Expression Is Regulated by FO in Huh7 Cells

**Figure 3:**
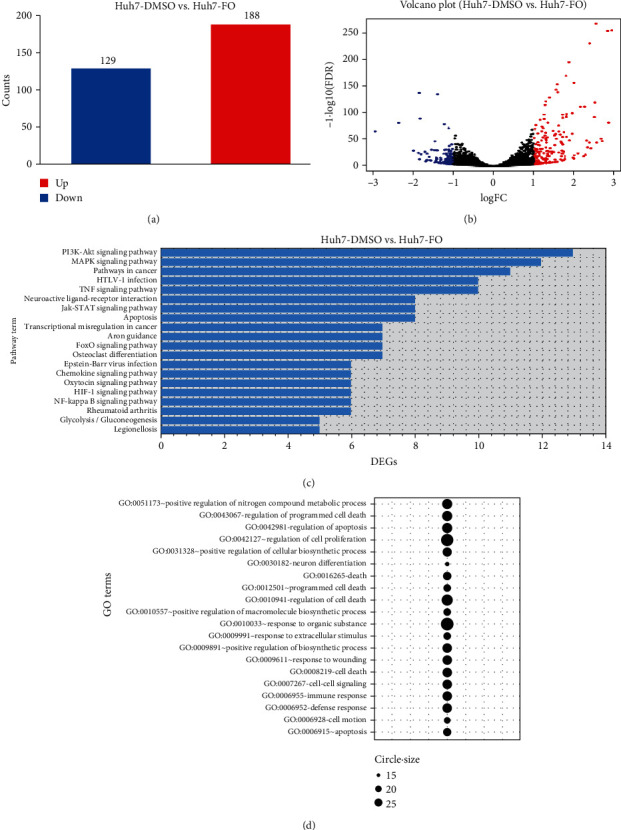
Bioinformatics analysis in FO-treated Huh7 cells. (a, b) Volcano plot of the differentially expressed genes. The thresholds were log2FC > 1 and adjusted *P* value < 0.05. Red dots indicate upregulated genes, and blue dots indicate downregulated genes. (c) Top 20 KEGG pathways with the most enriched differentially expressed genes of RNA-Seq (*P* < 0.01). (d) Bubble plot represents the top 20 gene sets with the most enriched differential genes in the DAVID GO BP DIRECT annotation database obtained by comparing gene expression between Huh7 treated with FO and controls. The size of these bubbles represents the number of differentially expressed genes, assigned to the GO BP terms.

Based on previous data, we found that FO has a more obvious inhibitory effect on Huh7 cells than other HCC cells. Therefore, we chose Huh7 cells as a representative and performed RNA-Seq sequencing. An RNA sequencing study was used to screen the DEGs in FO- (2.00 mM) interposed HCC cells. Screened with the threshold of ∣log2(FC) | >1.00 and *P* < 0.05, 188 upregulated DEGs and 129 downregulated DEGs were identified from our RNA-Seq data (Figures 3(a) and 3(b)). The number of altered transcripts has been organized based on the log2FC or the *P* value. RNA-Seq data is shown in the supplementary material.

### 3.3. Bioinformatics Analysis

KEGG analysis was performed to identify the most significant pathways regulated by FO, and the data showed that DEGs were generally enriched in signal pathways such as PI3K-AKT (*P* = 2.28*E*‐04), apoptosis (*P* = 3.19*E*‐04), MAPK (*P* = 6.17*E*‐05), and JAK-STAT (*P* = 6.77*E*‐04) (Figure 3(c)) pathways. To determine which biological processes (BP) can be regulated by FO, GO analysis was performed and the data was shown as a bubble plot (Figure 3(d)). The GO BP analysis revealed that the following DEGs significantly enriched in GO terms were involved: “GO: 0042127 regulation of cell proliferation” (*n* = 24, *P* = 0.007), “GO: 000726 cell-cell signaling” (*n* = 20, *P* = 0.006), “GO: 0006915~apoptosis” (*n* = 17, *P* = 0.04), and “GO: 0051726 regulation of cell cycle” (*n* = 12, *P* = 0.02).

**Figure 4:**
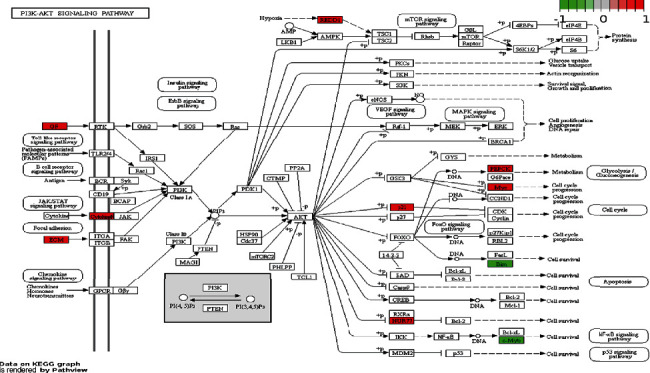
PI3K-AKT signaling pathway in Huh7 cells treated with FO. Expression changes of target genes are mapped by colors: red color marks increased genes; green color marks decreased genes.

**Figure 5:**
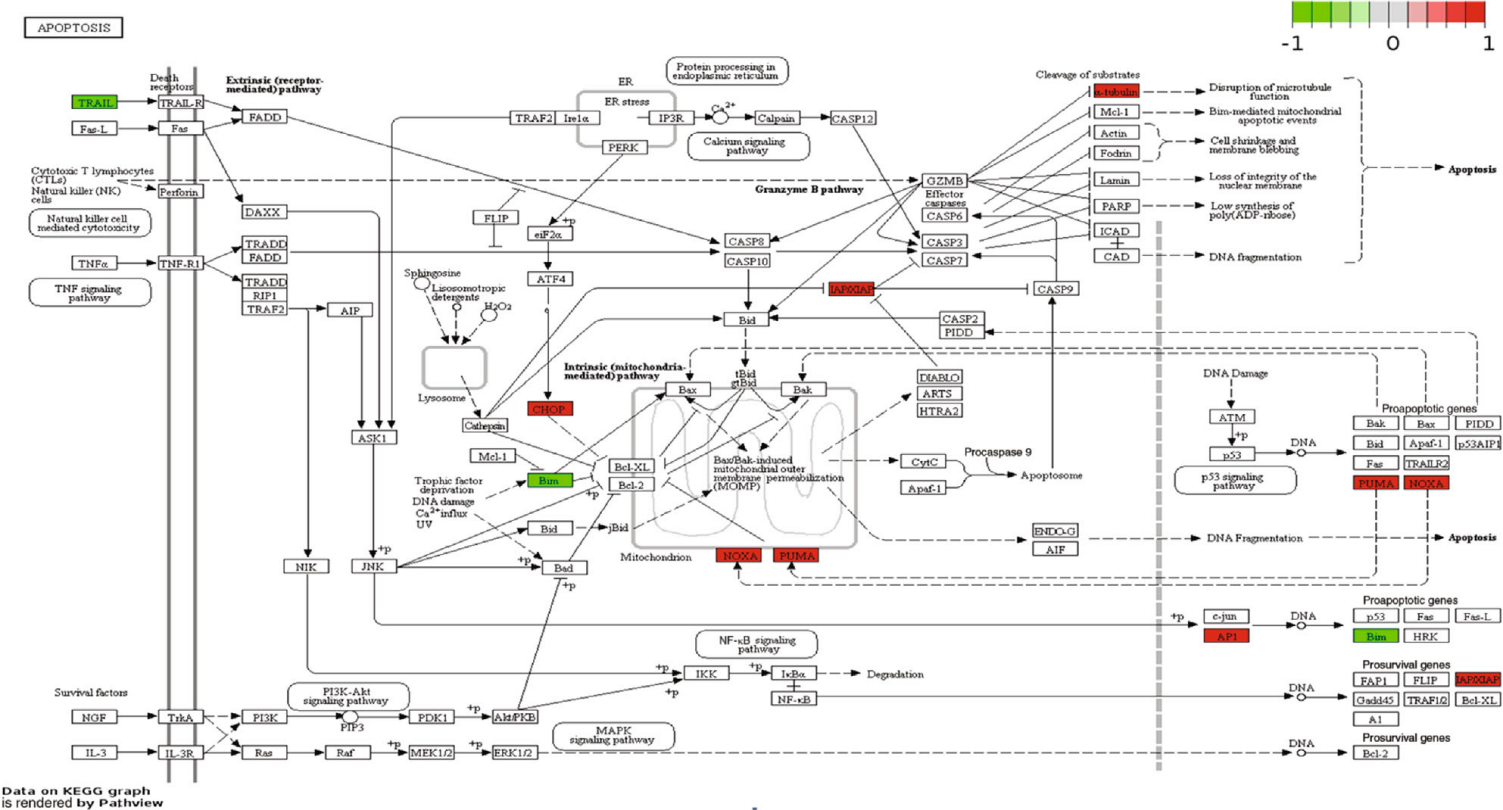
Apoptosis signaling pathway in Huh7 cells treated with FO. Expression changes of target genes are mapped by colors: red color marks increased genes; green color marks decreased genes.

The regulatory effect of FO on cell proliferation and apoptosis in critical signal pathways was confirmed by the Pathview library, and fold values of DEGs were mapped with appropriate colors for each gene forming the “PI3K-AKT signaling pathway” (Figure 4) and “Apoptosis” (Figure 5). The green color indicates downregulated genes, and the red color refers to upregulated genes. Analogous to the DAVID analysis with circosplot visualization, most of the genes displayed in “Apoptosis” and the “PI3K-AKT signaling pathway” were upregulated. These results suggest that FO may exert its antitumor ability by regulating key genes in these signaling pathways.

### 3.4. Screening and Verification of Candidate Genes

**Figure 6:**
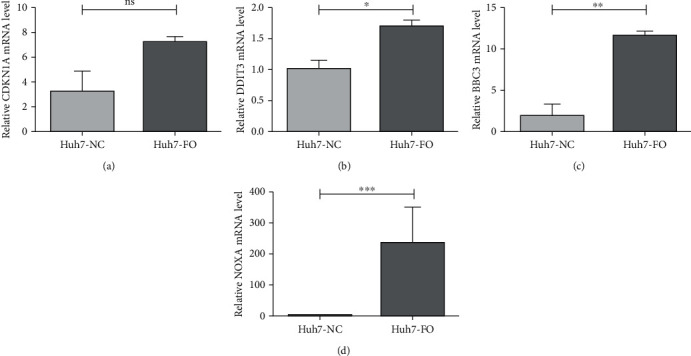
Real-time Q-PCR validation of RNA-Seq data. BBC3, DDIT3, CDKN1A, and NOXA. Results are presented as means ± SEM; *n* = 3. Statistical comparison by Student's *t*-test: ^∗^*P* < 0.05; ^∗∗^*P* < 0.02; ^∗∗∗^*P* < 0.01.

Combining the results of GO and KEGG enrichment analysis, the candidate genes that were recruited included not only those in the GO BP terms related to proliferation and apoptosis, but also those in the PI3K-AKT or apoptosis pathway. The hub genes consist of BCL-2 Binding Component 3 (BBC3) (log2FC = 1.407496566, *P* = 4.31*E*‐36), DNA Damage Inducible Transcript 3 (DDIT3) (log2FC = 1.67197457, *P* = 3.19*E*‐48), Cyclin-Dependent Kinase Inhibitor 1A (CDKN1A) (log2FC = 1.125627031, *P* = 7.16*E*‐28), and PMA-Induced Protein 1 (NOXA) (log2FC = 1.699459504, *P* = 1.41*E*‐29), which were also validated by Q-PCR and shown to be consistent with the RNA-Seq data (Figure 6).

## 4. Discussion

Clinically, unrestrained proliferation is the primary pattern of a tumor cell, and therefore, inducing apoptosis is the main strategy for tumor treatment [8]. Apoptosis consists of extrinsic signal pathways and intrinsic signal pathways. The former is mediated by cell surface death receptors, and the latter is initiated in mitochondria [9]. In the mitochondrial apoptotic process, the membrane permeability of mitochondria shows a reduction, and the level of BCL-2-related antiapoptosis factors is crippled [10]. In the BCL-2 family, the proapoptotic and antiapoptotic processes are concluded. The proapoptotic proteins induce mitochondrial release of cytochrome c, which plays a key role in activating mitochondria-dependent death, while antiapoptotic proteins work by preventing this release [11, 12]. In addition, cytochrome c promotes the formation of apoptotic multiprotein complexes by accumulating caspase 9 and apoptotic protease activating factor 1 (Apaf-1) in the cytoplasm [13]. Caspase 9 is an apoptotic promoter protease which can activate downstream caspase 3, then cleaves cellular substrates, and eventually induces cell apoptosis [14].

PUMA (BBC3) and NOXA (PMAIP1) are members of the BCL-2 family, and both respond to a variety of intracellular stress signals, such as hypoxia, losing of growth factors or cytokines, DNA damage, and anticancer drugs, regulating cell death [15]. BBC3 and NOXA are BH3-only proteins with proapoptotic activity, which can bind antiapoptotic proteins and inhibit their activities. In addition, they can also directly or indirectly affect BAK and BAX. The activated BAK and BAX can form oligomers and cause mitochondrial permeability to change. Subsequently, cytochrome c and second mitochondria-derived activator caspase (SMAC) are released from the interstitial space into the cytoplasm, and then induce apoptosis by activating caspase [16–20].

CDKN1A (p21), a cell cycle-dependent kinase inhibitor, has been identified as a target gene downstream of p53 and can promote apoptosis in many tumor types by activating the TNF receptor or inducing the proapoptotic protein BAX [21, 22]. Similar to PUMA and NOXA, p21 could also regulate cell apoptosis by mediating changes in mitochondrial membrane permeability. Interestingly, PUMA has also been proven to be an important factor in p53-mediated apoptosis and regulated by p21, which transmits death-related signals to mitochondria and promotes apoptosis [23–26]. Therefore, our results implied that p21 and PUMA might interact with each other to regulate cell apoptosis.

CHOP (DDIT3) is a member of the C/EBP transcription factor family, and its expression is related to endoplasmic reticulum stress (ERS) [27]. During ERS-related apoptosis, CHOP functions as an upstream factor regulating BCL-2 family proteins, such as PUMA [28]. In addition, CHOP-mediated cell death involves the induction of multiple genes that may enhance apoptosis, including ATF3 and TRIB3 [29, 30]. ATF3 is a transcriptional repressor that can trigger apoptosis, and TRIB3 inhibits NF-*κ*B activation, thus reducing survival. Our RNA-Seq data also showed that the expression of TRIB3 (log2FC = 1.593678323; *P* = 1.18*E*‐141) and ATF3 (log2FC = 2.838645787; *P* = 1.22*E*‐256) was upregulated in FO-intervened Huh7 cells. It implies that in addition to those hub genes, other unrevealed genes might also be targeted by FO. FO might be a multitarget drug accelerating tumor cell apoptosis.

**Figure 7:**
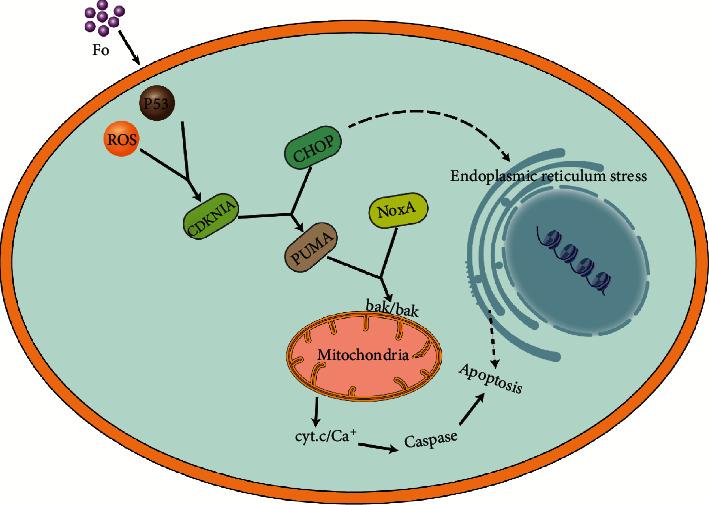
Schematic representation of the proposed model for the mechanism of FO-induced apoptosis.

Based on the above analyses, we simulated a potential signal pathway model of FO-induced apoptosis in HCC cells. FO might induce the production of reactive oxygen species (ROS) and activate p53, which will then enhance the level of CDKN1A. The upregulation of CDKN1A is often accompanied by the activation of PUMA and NOXA. Meanwhile, the upregulated CHOP might activate endoplasmic reticulum stress. Similarly, the elevating of CHOP also mediates the expression of PUMA and NOXA. Therefore, we speculate that PUMA and NOXA may be the key targets downstream of this signaling pathway and are both regulated by CDKN1A and CHOP. All the above altered proapoptotic factors might activate BAX-forming oligomers and might transfer from the cytoplasm to the mitochondria. Increased BAX changes the mitochondrial permeability and releases cytochrome c and calcium ions (summarized in Figure 7). The accumulated Ca^+^ in the cytoplasm will further activate the caspase cascade and eventually lead to apoptosis. But, the signal pathway model is only a hypothesis based on our bioinformatics analysis and other research reports. Therefore, in the future, some molecular biology researches and patient samples will be conducted to confirm this specific model on the FO-related apoptosis pathway.

## 5. Conclusion

We provide evidence that FO can inhibit proliferation and stimulate apoptosis in Huh7 and Hep3B cells. Moreover, the critical pathways and potential target genes modulated by FO were screened. The conceptual diagram of the signal that FO stimulates apoptosis by regulating hub genes is simulated, but the specific mechanism by which FO mediates signal pathway transduction by regulating the expression of candidate genes is still unclear. We also need to reveal this hypothesis in subsequent molecular biology and histopathological experiments. It implies that the reformed FO can inhibit tumors by their effect on genes in critical signals and can be used as a promising antitumor drug.

## Data Availability

The data used to support the results of this study are included within the research.

## Abbreviations

FO: N-*trans*-Feruloyloctopamine
HCC: Hepatocellular carcinoma
Q-PCR: Quantitative polymerase chain reaction
KEGG: Kyoto Encyclopedia of Genes and Genomes
DEGs: Differentially expressed genes
PBS: Phosphate-buffered saline
DMSO: Dimethyl sulfoxide
GO: Go ontology.
